# BOWS (bioinformatics open web services) to centralize bioinformatics tools in web services

**DOI:** 10.1186/s13104-015-1190-0

**Published:** 2015-06-02

**Authors:** Henrique Velloso, Ricardo A Vialle, J Miguel Ortega

**Affiliations:** Departamento de Bioquímica e Imunologia, Instituto de Ciências Biológicas, Universidade Federal de Minas Gerais (UFMG), Belo Horizonte, MG Brazil

**Keywords:** Web services, Bioinformatics, HPC

## Abstract

**Background:**

Bioinformaticians face a range of difficulties to get locally-installed tools running and producing results; they would greatly benefit from a system that could centralize most of the tools, using an easy interface for input and output. Web services, due to their universal nature and widely known interface, constitute a very good option to achieve this goal.

**Results:**

Bioinformatics open web services (BOWS) is a system based on generic web services produced to allow programmatic access to applications running on high-performance computing (HPC) clusters. BOWS intermediates the access to registered tools by providing front-end and back-end web services. Programmers can install applications in HPC clusters in any programming language and use the back-end service to check for new jobs and their parameters, and then to send the results to BOWS. Programs running in simple computers consume the BOWS front-end service to submit new processes and read results. BOWS compiles Java clients, which encapsulate the front-end web service requisitions, and automatically creates a web page that disposes the registered applications and clients.

**Conclusions:**

Bioinformatics open web services registered applications can be accessed from virtually any programming language through web services, or using standard java clients. The back-end can run in HPC clusters, allowing bioinformaticians to remotely run high-processing demand applications directly from their machines.

## Background

Bioinformatics increasingly must deal both with large amounts of data provided by massive DNA sequencing efforts and with the novel patterns exposed by myriad systematic approaches. These data demand diverse and powerful software tools. Such tools are available, but users often face a wide range of difficulties as they attempt to get these tools running and producing results. There are often complex installation procedures, and many of these tools demand high processing power, which might not be available. Furthermore, each application has its own learning curve, forcing users to spend time learning how to use each one.

Bioinformaticians benefit from systems that centralize the tools needed for their work, using an interface for input and output. Web services, due to their universal nature and widely known interface, constitute a very good option to achieve this goal. Furthermore, web services provide a security layer that can restrict access to tools and protect copyrighted programs running on the back-end.

Bioinformatics centers are present all around the world, such as GenomeNet that mantains KEGG [1], UniProt consortium [2], SIB that mantains the ExPASy portal [3] and National Center for Biotechnology Information (NCBI). These and other centers offer tools which demand high performance computing (HPC). Integration of web portals with HPC is common nowadays. Thus, the availability of HPC applications might support the development of new portals.

Research activities in bioinformatics have great demands for services. Presently, virtually any biologist makes use of web servers to access bioinformatics applications, either accessing a specific tool or a portal. Conversely, the web services concept differs from services offered by such web servers that are designed for human access. Despite the enormous benefit given by the access to bioinformatics resources via web pages, there is also a demand for application programming interfaces (APIs) that can be accessed by scripts and are often incorporated into automated pipelines [4]. There are several major servers that provide access to various data resources and analysis tools like: European Bioinformatics Institute (EMBL-EBI) [5], NCBI E-utilities [6], Virginia Bioinformatics Institute (VBI) [7], Kyoto Encyclopedia of Genes and Genomes (KEGG) API service [1] and the DNA Data Bank of Japan (DDBJ) web API for bioinformatics [8]. A comprehensive collection of web services has been integrated in TogoWS SOAP and REST APIs [9]. Also, many other services can be found in BioCatalogue [10]. Different methods can be incorporated together with workflow engines such as Taverna [11], Kepler [12], or Galaxy [13] and access to them can be made available by web services. Recently, workflow portals such as Biowep [14] (Workflow Enactment Portal for Bioinformatics) and BioVeL [15] (Biodiversity Virtual e-Laboratory) were organized providing browser-based interfaces and also web services interfaces for remote invocation. Moreover, a comprehensive workflow repository is available in http://www.myexperiment.org [16].

Here we present bioinformatics open web services (BOWS), a web services platform that allows centralized and standardized access to bioinformatics tools. The major advantage of BOWS is that it is installed in an intermediary machine and provides a front-end to consumers of bioinformatics tools and a secure back-end to owners’ tools. The front-end exposes a WSDL (the document in XML or *Web Services Description Language* which describes a web service) with three web methods used by bioinformaticians to submit processes, check statuses and get results from applications. The back-end consists of five secure web methods used by tool owners to register new applications, delete existing applications, read new processes, change their statuses and submit results. This model creates a cyclical process where submissions are sent to BOWS via front-end services and then asynchronously executed by the back-end server. When the results are ready they are written back to BOWS and made available to the requester. The BOWS platform is not intended to replace other available resources as a global system, but instead aims to deliver by command line, GUI clients or web portal pages the access to asynchronous services executed under powerful HPC instances without bureaucracy. Thus, programmers that install applications with many dependencies, or that couple several programs in a pipeline, or that aim to protect the access to their code, can promptly give an API access “in-house” to their programs. Moreover, BOWS automatically generates a website that presents its registered applications and additionally, generates Java clients for submission of jobs and retrieval of results. Still, BOWS automatically writes a Java program to govern the HPC transactions with the system.

## Results

### System description

**Figure 1:**
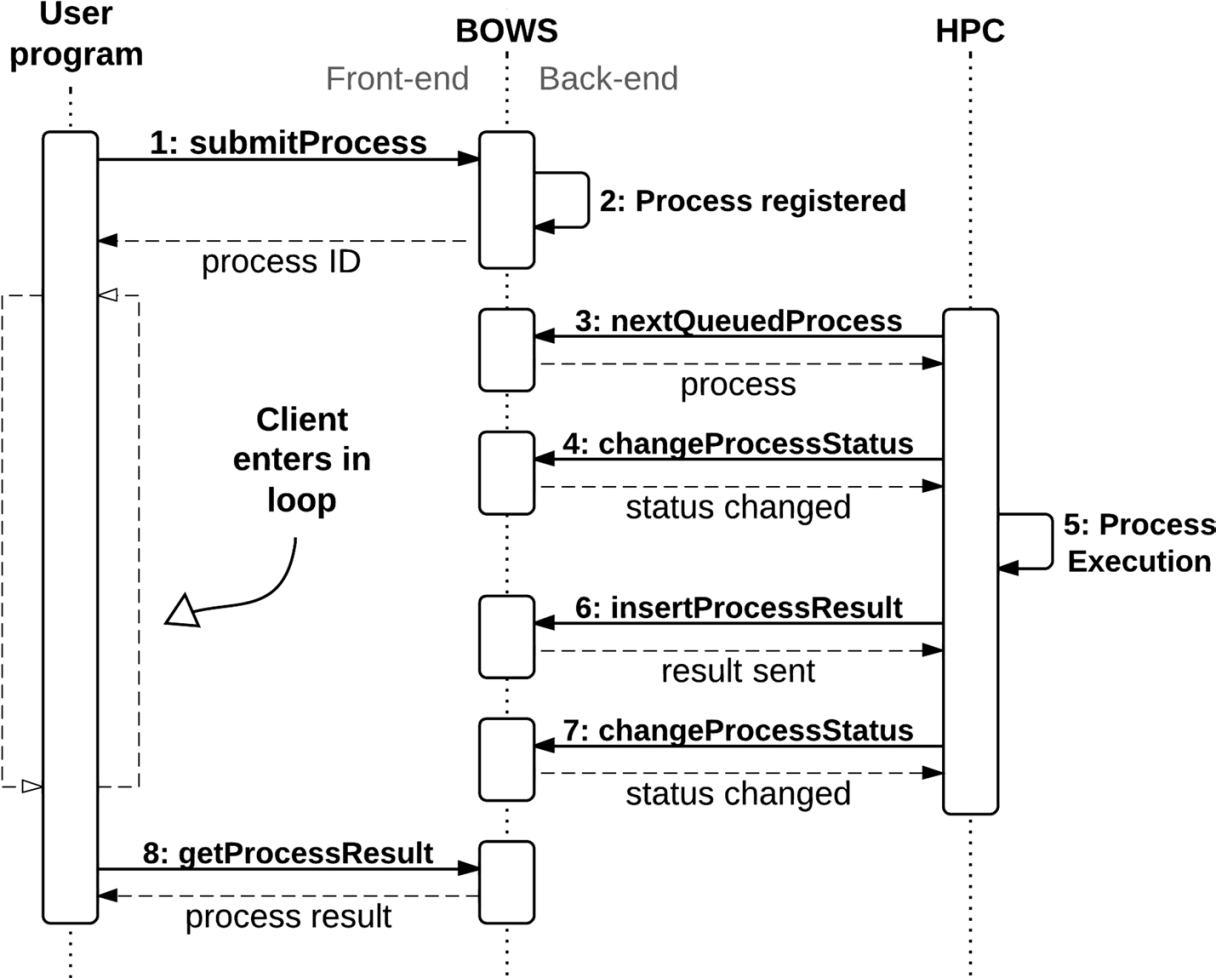
Sequence diagram showing the cycle of execution in BOWS. Front-end transactions are on the *left* while back-end transactions are shown on the *right*.

Bioinformatics open web services server offers two different WSDL files: a front-end WSDL and a back-end WSDL (Figure 1). Front-end transactions are made by client programs to submit new processes to registered applications and read results. Back-end transactions are performed by HPC instances to retrieve submitted processes and insert results after execution. Back-end transactions shall be used first, by programmers which will register their applications in BOWS platform.

Since intensive computing is often required by Bioinformatics applications, BOWS was designed to deal with asynchronous web service requisitions. To add high performance computing to the system through a safe and simple mechanism, requisitions are received by the BOWS front-end service and stored in a local database. An agent in the computational cluster named “arrow” is responsible for connecting with the back-end web service. To allow back-end transactions, BOWS makes available a restricted back-end web service with three web methods. The “arrow” agent accesses the back-end web service to look for new jobs using the nextQueuedProcess method. If one is found, the agent modifies the status from “queued” to “running” with the changeProcessStatus operation and processes the input. When the result is available, it saves the result back to BOWS using the insertProcessResult web method and changes the status to “finished”. If a processing error is found, the status can also be changed to “error”.

Safety is ensured because the cluster connects unidirectionally to the BOWS server, thus it is not necessary to request that systems administrators change any vulnerable setting, such as opening additional ports. Set up is simple since both the application and back-end connecting agent are written in any computational language. Crontab triggers the agent periodically (typically every minute). Therefore, the number of nodes typically required by the application is defined by the programmer, in the “arrow” agent. Virtually the HPC user does not require any special management to make the application to be linked to BOWS.

The back-end services should also be used to register a new application to BOWS. There are two administrative methods created to allow application owners set up and/or remove a new application: createApplication and removeApplication. Thus, this step up should be performed before executing processes.

Bioinformatics open web services front-end service provides web methods that allow users to submit processes to registered applications and read results. As the responses are asynchronous, typically the user program should enter a loop waiting for the availability of a process result. In general, the user program should operate as follows to access a tool in the BOWS platform:

1. Submit a new process with the desired parameters calling the front-end web method submitProcess.
2. Enter a loop where, at each iteration, it should check the status of the submitted process by calling the method checkProcess.
3. If the process status is FINISHED, it should call the method getResults to obtain the results.
4. If the process status is ERROR, it should handle the error.

### Installation

Bioinformatics open web services is available in http://sourceforge.net/projects/bows/. Files made available comprise a “user guide for BOWS installation and usage”, “bows.sql” that corresponds to BOWS database dump and two files “BOWS.war” and “BOWSWeb.war” which are intended to be copied into “webapps” folder of Apache Tomcat. “BOWS.war” provides the web services functionalities and WSDL file, while “BOWSWeb” builds a website interface. The user guide also contains example GUI programs to access BOWS services.

### BOWSWeb

In order to facilitate the management of the applications, a website interface called BOWSWeb (1) extracts information from BOWS database and presents information of all registered application, (2) includes a back-end transaction interface for creating and deleting applications and (3) automatically creates java clients for back-end transactions (“arrow” script) and front-end transactions (for either submitting jobs or for getting results). These features are explained below.

**Figure 2:**
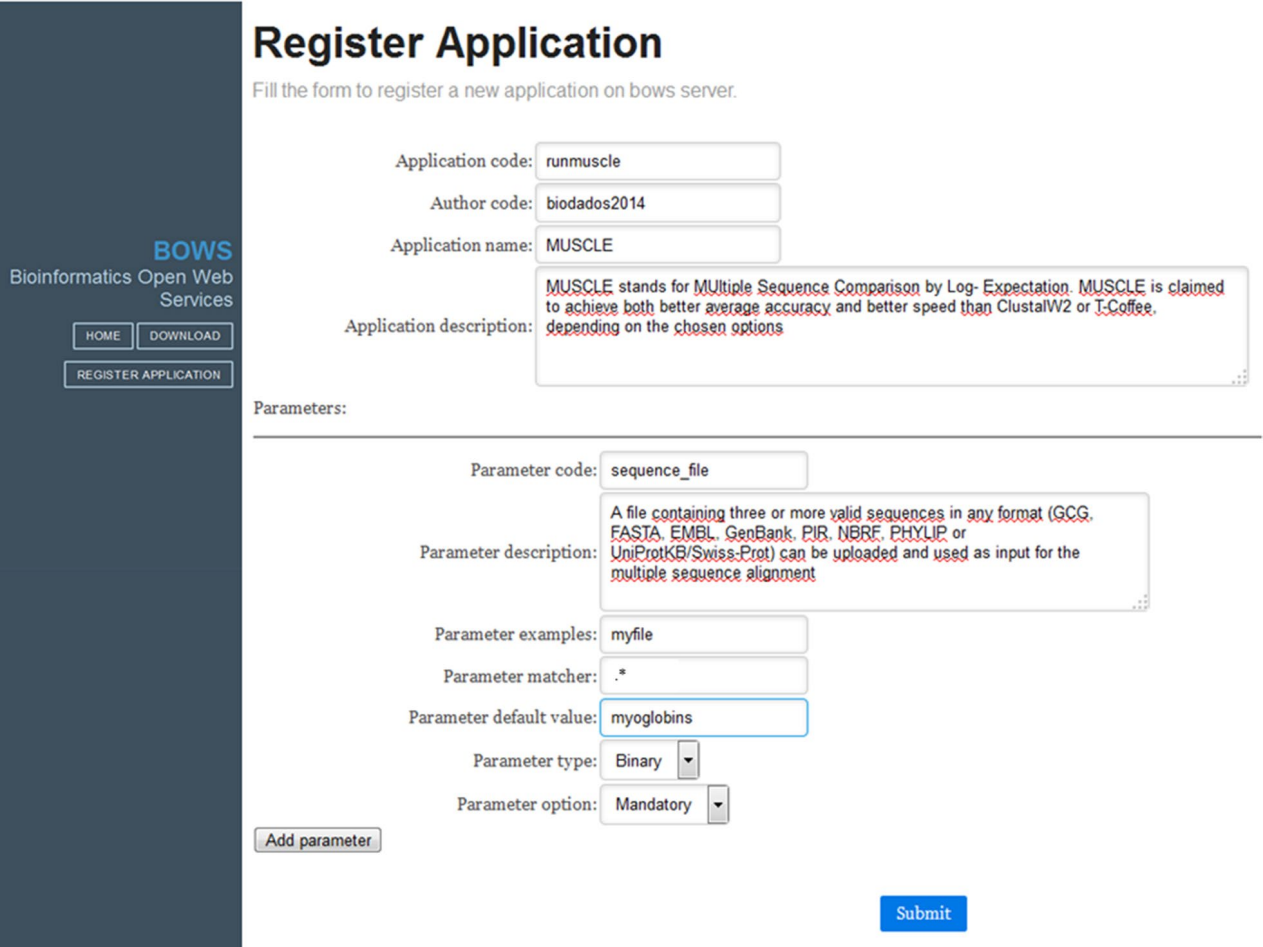
How to register an application in BOWS. This can be done by a webservice transaction or by filling this form in BOWS services website. In this example, a Submit Job client for running MUSCLE would be automatically compiled and disposed in the website (see the *green button* in Figure 3). To run the application, the command line would be: java-jar runmuscleSumitJob.jar-sequence_file < myfile > -user < name > . A link for creating the “arrow” program would appear (*red button* in Figure 3). It downloads a base “arrow” (Arrow_runmuscle.jar), a shell script (Arrow_runmuscle.sh) to complement the base “arrow” by informing the application execution command line in it, and a shell script to edit cron scheduler to execute the “arrow”.

To register an application, the method createApplication is called. This method requires, besides the information required to run the application (e.g. name, code, and parameters) the inclusion of an author code. Therefore, only the creator can subsequently delete a registered application. Registration can be done directly through the SOAP method (by using the message protocol *Simple Object Access Protocol* available for most programming languages), or more conveniently by using a BOWSWeb form interface. Similarly, to remove an application, the method “removeApplication” is called and there is an option to remove it in the website by just clicking a button. Both procedures require the author code to allow the exclusion. Figure 2 exemplifies how to register a new application in BOWS.

**Figure 3:**
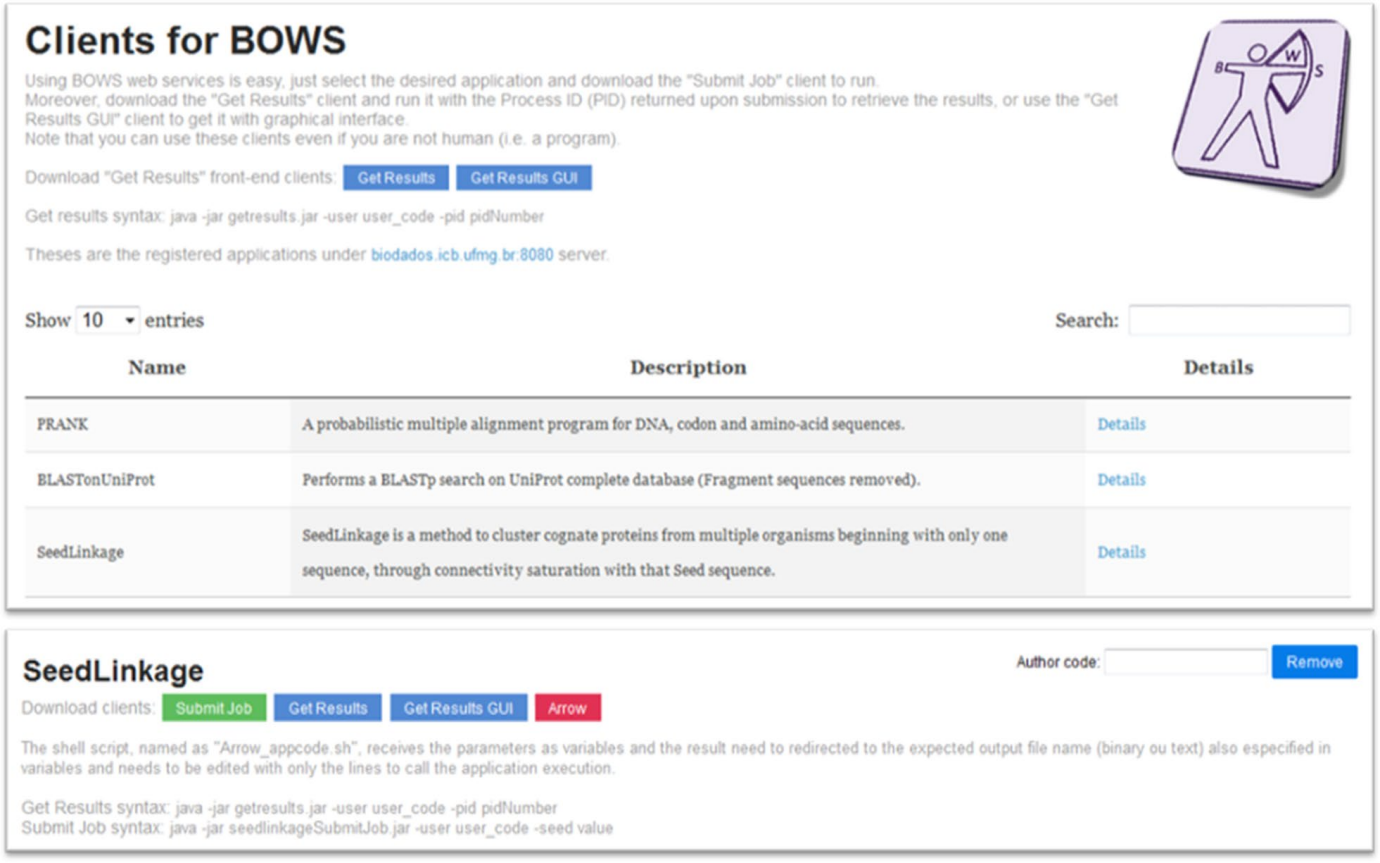
A view of BOWS services website. Whenever an application is registered in BOWS, the information provided is used to create a website with access to all applications (*upper panel*). A click on “Details” opens a page with access to the Java client used to submit jobs, command line and GUI clients to check status and retrieve results, a draft of the “arrow”, the program that controls the application in the HPC (*lower panel*) and the description of the parameters required (not shown).

Using the information registered for each application BOWSWeb displays in a website the information on all registered applications, as exemplified in Figure 3. Moreover, with the information available in its database (e.g., parameter names and types), BOWSWeb automatically generates java clients to call BOWS methods hidden from the final user. Clients are generated for both the front-end and the back-end web services and are distributed by the website. Therefore, users do not even need to learn how to use SOAP protocols.

For the front-end two executables are created, one for job submission and one for the results retrieval (bottom panel in Figure 3). The submission client name follows the pattern “appcodeSunmitJob.jar” and receives as arguments the pre-defined parameters called with flags tagged by the parameter names. If the expected parameter is a binary file only the file name is required, and the client will load the file by itself. Also an additional parameter “user code” called by the flag “-user” is required to protect that anyone may visualize the results when available. Therefore when a job is submitted the SubmitJob returns a process id that should be used together with the user code on the results retrieval client. Figure 3 shows a green button that is a link to download the client for the application SeedLinkage. The “getresults.jar” client is the same for any application, it receives only two parameters: the process id (using the flag “-pid”) and the user code (using the flag “-user”). If the process result is a text, it will be show on screen; otherwise a file named after the pid will be saved. It is also downloadable from the website.

For the back-end three files are generated, the java executable “arrow”, one shell script which will link the “arrow” with the application and another shell script that configures cron to execute the “arrow”. The shell script, named “Arrow_appcode.sh”, is expected to be edited by adding the command line needed to call the application execution. Then, the java client, named “Arrow_appcode.jar”, manages the SOAP requisitions to look for new submitted jobs, execute the application calling the “Arrow_appcode.sh” and send the results to BOWS server. The java client needs to be kept running to process the jobs, we recommend using a crontab schedule, and to facilitate the installation we provide a script called “add_to_crontab.sh” that automatically configures this feature. Although expert programmers might be able to program the “arrow” cycle, this feature of BOWSWeb may prompt BOWS usage. A red button in the website (Figure 3, bottom panel) is a link to download a zip file containing the “arrow” and the script.

BOWSWeb also distributes in the website a java getresults client with graphical interface. The “GetResultsGUI.jar” is a java executable that shows a simple interface showing the boxes for the process id and the user code needed to retrieve a result. It also allows the user to save the file where desired. The same idea can be applied for the submit process, so the distribution of GUI clients to colleagues can facilitate the use of applications on any machine that has java. Webpages also can be created by local research groups where the submission of requests is functionally similar to these GUI clients.

Thus, BOWSWeb lists all applications registered in BOWS, allows for an easy registration of a new application, generates the specific java client for submitting a job to this application, provides a general java client to retrieve results and, remarkably, generates a java program to control the HPC connection through the back-end. Therefore, any program installed in a HPC can be promptly accessed as an API, not only by SOAP protocol, but also by executing the java client generated by BOWSWeb.

### Case studies

Three case studies were conducted in the BOWS platform and are available for tests at http://biodados.icb.ufmg.br/bows (Figure 3).

The multiple sequences aligner Prank [17] was registered in an online BOWS server. The client receives as parameter an input file in MULTIFASTA format and submits a process to the BOWS platform registered as “PrankAlign”. The demo client uses the three methods in a row: submitProcess, checkProcess and getResults, in a transparent manner. Results show the multiple alignments performed in BOWS platform. It is important to note that on the client machine all that is required is the installation of the Java virtual machine. The user of this client is not aware of which web server executed the multiple sequences alignment. Remarkably, “user” might be a script. Execution is as simple as: *java* -*jar prankalignSubmitJob.jar* -*fasta myoglobins* -*user userCode*.

The second application is a very well-known one, BLASTp [18], however it searches an UniProt database comprised of only “Complete” sequences (depleted of “Fragments”). This is an example of a case where a common application is used, however it searches a database periodically maintained by a remote group.

**Figure 4:**
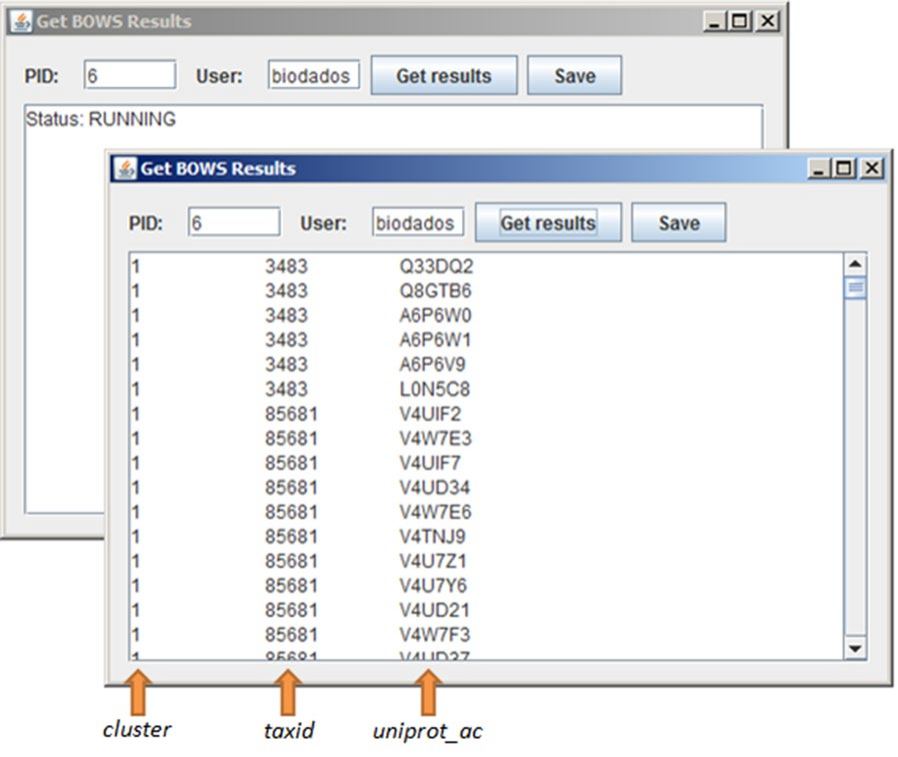
Graphical User Interface Get BOWS Results. A front-end client can be used to check the status (window in *second panel*), retrieve, and save results. In this case, the program Seed Linkage created a cluster of orthologues for THC synthase (uniprot_ac Q33DQ2) from *Cannabis sativa* (taxid 3483), finding sequences from *Citrus clementina* (taxid 85681). Seed Linkage has many dependencies and is thus hard to install, but it was easily executed with the Java client downloaded from BOWS website with the command line: java-jar seedlinkageSubmitJob.jar-seed Q22DQ2-user biodados.

The third example is SeedLinkage [19], a software produced by our group that creates a group of orthologues related to a Seed. This example was included because SeedLinkage installation is painful due to several dependencies. Therefore, by using BOWS, it can be executed remotely with just a java command line. Figure 4 shows the GetResultsGUI.jar interface. First execution retrieved the information “RUNNING” and, when the job was completed, the interface shows the cluster number, the taxonomy identifier of the clustered sequences, and the UniProt accession for the grouped proteins. By pressing the Save button results can be conveniently stored. In the sourceforge distribution, submit job clients specific for these examples were included.

### Expected usage

Several groups working on bioinformatics develop software or pipelines and there is often a lag until these applications can have an application programmatic interface (API), at which time other colleagues and collaborators can promptly add them as routines or methods in distinct programs. Moreover, frequently the research group works with a pipeline in which just a few parameters are changed, aiming to preserve the characteristics of the approach taken by the group, so it is convenient to distribute amongst colleagues and collaborators simple Java programs that will process the data with a few specific parameters while the rest of the pipeline follows the original set up. To use BOWS it is necessary that a programmer with large expertise installs the application and edits the “arrow” script, which accesses BOWS from the back-end. However, after registering the application, BOWS not only provides software access with the front-end methods, which require the knowledge of SOAP, but automatically generates a BOWS clients website which contains Java programs, parameters description, and default examples. Front-end users can run those Java programs or, although not so elegant, write scripts in other programming languages such as Perl or Python and execute the Java programs within them. Thus, BOWS may be used to provide global access to in-house software through a very simple procedure, protecting access to the HPC that executes the application and keeping its code. However, its main usage might be to quickly provide software and simplified command lines to colleagues that share HPC access. BOWS shortens the gap between installation of an application and sharing it, without exposing the HPC to direct access.

Virtually, any tool can be used with BOWS, although with this version both input and output might be restricted to 4 Gb, because this is the limit that can be populated in the intermediary MySQL database. However, both input and results can be delivered by sending the file location through BOWS, as a parameter. Bioinformatics, which characteristically deals with software with complex dependencies, thus would find BOWS very useful; however, its use for research on other areas such as chemistry, physics, etc., is also possible.

### Drawbacks and limitations

One important drawback is that the transactions are based on SOAP. A version might be available in the future based on Json, so the requisitions can be executed by REST. This will facilitate the usage by programs since the lag for learning how to code a Json object is relatively short. Although the encapsulation of transactions in Java clients provides prompt usage of the platform, results obtained still require parsing, thus pushing the application programmer to provide them in a well-designed format. The most important drawback might also be its greatest feature: the access to HPC power can be extended to unregistered users. Once the application is installed in the HPC by a registered user, its access is extended to colleagues. Thus, this registered user might be overloading the HPC. To deal with this, application author must limit the number of instances/nodes/cores used by the application, since BOWS automatically will send just one proves at the time, when the nextQueuedProcess method is called.

A limitation might be not to provide data and procedures reuse if they are produced in the HPC, and data provenance (tracing and recording the origins of data and its movement). One possible approach is the expert programmer to split the workflow in independent applications, and then the front-end programmer will execute them as routines or methods, controlling data and procedures reuse, and provenance. This is a limitation because two front-end users must contact each other to share intermediary results.

## Conclusions

BOWS provides a wide range of benefits to both Bioinformatics tools consumers and owners. The Web methods are generic and use a common syntax to access any registered application, so users only have to learn once and are ready to use any tool. Also, due to the universal nature of web services, BOWS registered applications can be accessed from virtually any programming language, as most of them provide extensive support to build web service clients. This solution also protects the code and intellectual property of applications, since the final users do not have access to the code or the binaries. Finally, the back-end can run in HPC clusters, allowing bioinformaticians to remotely run high-processing-demand applications directly from their machines, which otherwise would be unfeasible. Installation files and instructions for the BOWS platform are available in sourceforge.net. BOWSWeb facilitates the usage, providing BOWS with a compelling interface.

## Availability and requirements

Project name: BOWS

Project home page: https://sourceforge.net/projects/bows

Operating system: Linux

Programming language: Groovy, Java

Other requirements: Java 6 or higher, Apache Tomcat 7 or higher, MySQL 5.0 or higher.

License: BSD

Any restrictions to use by non-academics: None

## Abbreviations

BOWS: bioinformatics open web services
HPC: high-performance computing
